# Dorsal root ganglion stimulation provides significant functional improvement from acute debilitating Crohn's disease: A novel use

**DOI:** 10.1016/j.inpm.2024.100389

**Published:** 2024-02-02

**Authors:** Harman Chopra, Melissa Jackels, Michael Suarez, Peter D. Vu, Mustafa Broachwala, Tariq AlFarra, Eellan Sivanesan

**Affiliations:** a Johns Hopkins University School of Medicine, USA; b Nazareth Hospital, USA; c The University of Texas Health Science Center at Houston, USA; d Icahn School of Medicine at Mount Sinai, USA

**Keywords:** DRG, Crohns, IBD, Neuromodulation, Acute pain crisis, IBD flare, Neuropathic pain

## Abstract

Crohn's disease is a chronic inflammatory bowel condition causing symptoms, notably pain, due to ongoing intestinal inflammation or complications like abscesses, strictures, and fistulas, which are common in IBD patients. Abdominal pain affects up to 60 % of IBD patients, irrespective of disease severity, prompting medical attention. Various medications like NSAIDs, antidepressants, antispasmodics, anticonvulsants, and opioids are used to manage pain, but they have limited effectiveness and potential side effects, even during remission. In this case, a 20-year-old Caucasian female college student [height 5′4″, weight 120lbs (54.4 kg)] with juvenile idiopathic arthritis and Crohn's disease experienced severe daily abdominal pain, negatively impacting her life. Despite a multimodal regimen, including gabapentin, nortriptyline, duloxetine, and acetaminophen, her pain persisted, significantly affecting her appetite, sleep, mood, activity level, and overall quality of life (QOL). To address this, dorsal root ganglion (DRG) stimulation was considered. The patient aimed for a 20 % pain reduction and improved QOL. Trial leads were placed along the T10 and T12 DRG, resulting in a 25 % pain reduction (8–6 out of 10) and substantial QOL improvement. She could eat, sleep without interruptions, walk longer distances, and be more active. The T12 lead was more effective than the T10, targeting upper abdomen stimulation. The patient and her mother were highly satisfied and opted for permanent implantation for the T11 and T12 DRG. While DRG stimulation was approved in 2016 for chronic pain, to our knowledge, this is the first reported case of its use in a patient with debilitating Crohn's disease.

## 1 Introduction

Inflammatory bowel disease (IBD) affects more than 3 million people in the United States, with over 70,000 new cases diagnosed each year [1]. Crohn's Disease (CD) is a type of IBD that can affect any area of the gastrointestinal tract, causing chronic inflammation and disruption of the intestinal mucosa, with periods of flares and remission [2]. The most prevalent symptom of CD is abdominal pain, which can occur regardless of the disease activity or remission status [3]. Studies have shown that 20–50 % of CD patients in remission still suffer from pain, and that abdominal pain is a common complaint among IBD patients throughout their lives, irrespective of disease severity [4].

Pain in the setting of CD may be multifactorial in nature, and existing literature suggests not only active inflammation, but also secondary complications such as abscess, stricture, intermittent small-bowel obstruction, bile-acid malabsorption, and functional pain, contribute to symptoms and decreased quality of life reported in this patient population [3]. There are many treatment modalities for CD pain including pharmacologic and non-pharmacological interventions [3,5]. The current pharmacological options and mainstay in treatment for CD pain primarily target inflammation but may not be effective for non-inflammatory pain [5]. These include immunomodulators, steroids, and monoclonal antibodies [6]. Other pharmacological agents, such as antispasmodics, NSAIDs, laxatives, antidepressants, antiemetics, COX-2 inhibitors, cannabis, and opioids have been used for CD pain, but have limited efficacy and/or significant adverse effects. Non-pharmacological interventions, such as cognitive-behavioral therapy, acupuncture, and hypnotherapy may offer some benefits, but their availability and accessibility are often limited [5].

A major factor that drives IBD patients to seek medical care is pain. Compared to non-IBD patients, CD patients have higher annual admission rates, three times higher direct healthcare costs and more than double the out-of-pocket expenses [7]. The average lifetime incremental cost of CD is over $400,000, regardless of the age of diagnosis [1]. These numbers underscore the urgent need for cost-effective strategies to address the challenges faced by patients and families affected by IBD. Unfortunately, there is no overarching consensus among healthcare providers on the optimal therapy for CD related pain. Given limited evidence on the comparative effectiveness of different therapies, ongoing research is needed to identify optimal treatment options that are both successful and cost-effective. We present a compelling case of a severe and debilitating acute on chronic pain crisis in the setting of Crohn's disease and showcase, to our knowledge, a novel use of DRG stimulation.

## 2 Case report

**Fig. 1:**
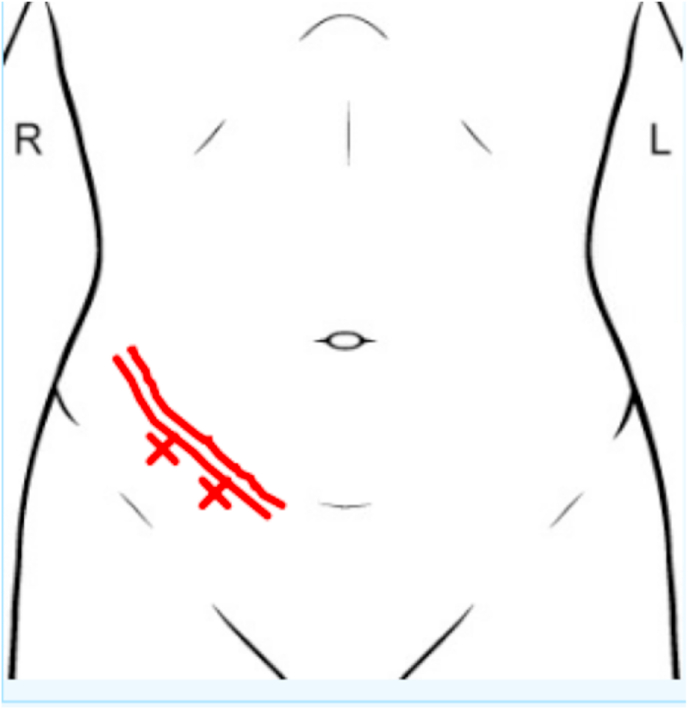
Pain distribution described by patient on initial encounter.

A female college student with a past medical history of Juvenile Idiopathic Arthritis and Crohn's Disease was referred for evaluation of chronic debilitating abdominal pain for 2-years, starting around the time of her Crohn's diagnosis. She reported a persistent dull aching sensation in her lower right abdomen that fluctuated in severity (Fig. 1). Using the numerical rating scale (NRS) ranging from 0 to 10 [8], she reported typical pain as a 5–6/10 but reported occasional episodes of 9/10 in intensity. The pain was described as throbbing and aching in quality, without radiation outside of the abdomen. She experienced severe pain shortly after ingesting food which would persist for several hours. The patient reported that foods high in fat, fiber or with abrasive qualities aggravated the pain more than others, but almost all food caused discomfort.

The patient required a peripherally inserted central line (PICC) line for supplemental feeds after a recent hospital admission for her pain, which was so severe she was unable to tolerate oral intake. Her medications included Ustekinumab infusions and a multimodal pain regimen of gabapentin 900mg TID, nortriptyline 10mg daily, duloxetine 60mg daily and acetaminophen as needed. These interventions did not result in improvement of her pain. Her pain severely impaired her appetite, ability to eat, sleep, mood, activity level and overall quality of life (QOL).

**Fig. 2:**
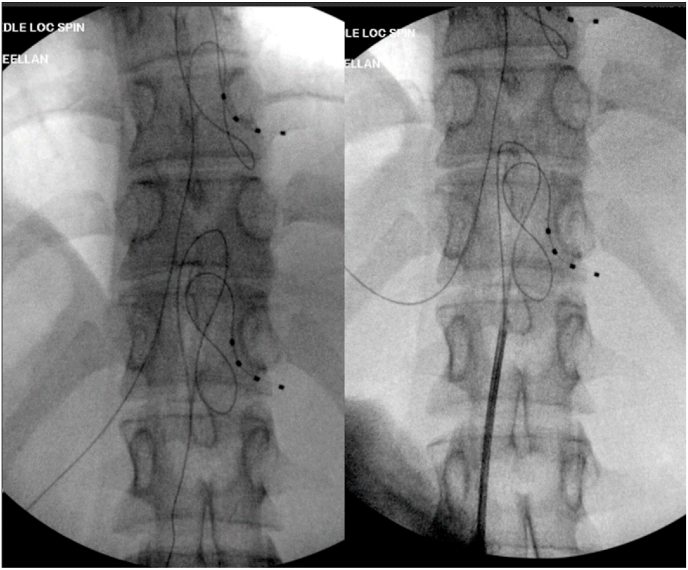
T10/T12 DRG trial lead placement.

The decision was made to trial DRG stimulation. The patient's desired outcome was to achieve pain relief, but primarily to be able to exercise, eat solid foods, and attend school. Trial leads were percutaneously placed under fluoroscopic guidance along the T10 and T12 DRG (Fig. 2). The procedure was tolerated well without complications and the patient was discharged home.

**Fig. 3:**
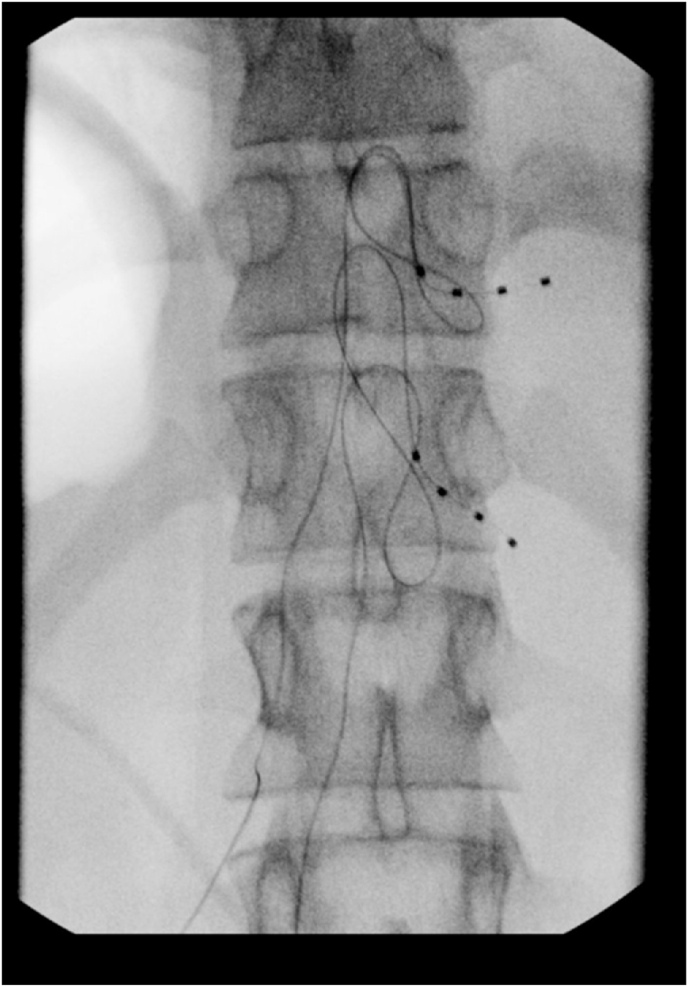
Lead placement at T11/T12 DRG for permanent implant.

Following the DRG stimulation trial, the patient's pain score decreased from 8 to 6 out of 10 (25 % reduction) at the follow-up visit. She reported a remarkable improvement in her quality of life, stating that she was now able to eat by mouth, had improved sleep and was not waking up during the night with significant pain. She also reported getting out of bed more frequently and ambulating longer distances. Overall, she preferred the T12 lead over the T10 lead. She stated that the lower lead (T12) worked better for her as the T10 lead provided stimulation in the upper abdomen that was less helpful. The patient and her mother expressed extreme satisfaction and wished to proceed with the permanent implant. A right T11/T12 DRG stimulator implant was placed approximately 4 months post-trial (Fig. 3).

Four days post-procedure, the patient reported symptoms of nausea, vomiting, fever (101.4°) and pain. She was diagnosed with Staphylococcus epidermidis bacteremia, and the source of infection was suspected to be her PICC line, which was subsequently replaced with a double lumen Hohn catheter for total parenteral nutrition (TPN) administration. A thoracic spine CT scan revealed a small fluid collection near the DRG stimulator, which was considered within the normal range of postsurgical changes. Neurosurgery was consulted and did not advise removal of the implant.

At her two-week follow-up visit, the patient continued to report a significant reduction in pain after the implant. Before the DRG placement, she reported constant pain of 8/10 intensity, which increased to 10/10 after eating. At this visit, she rated her pain as 5/10 and 5–6/10 after eating. She had no fever, chills, nausea, or other signs of infection systemically or at the incision site. She also began tolerating some soft foods orally at this time. At her four-week follow-up visit, she reported continued improvement in daily function and oral tolerance since her permanent implant. Her pain level was 5/10 (NRS) and manageable post-prandially. She was subsequently able to wean off TPN and attend her classes at school.

## 3 Discussion

As a chronic incurable disease, with most new cases occurring in patients younger than 35, CD imposes a high-cost burden on the healthcare system and on the quality of life of patients and their families [1]. Compared to non-IBD patients, CD patients have higher annual admission rates, three times higher direct healthcare costs and more than double the out-of-pocket expenses [9]. The average lifetime incremental cost of CD is over $400,000, regardless of the age of diagnosis. The lifetime total cost of CD is $622,056, which includes outpatient ($273,056), inpatient ($164,298), pharmacy ($163,722), and emergency room (ER) ($20,979) costs. Based on the prevalence of CD in the United States in 2016, the lifetime total costs for this population amount to $498 billion [1]. These numbers, which are likely to have subsequently increased, underscore the need for cost-effective strategies to address the challenges faced by patients and families affected by CD. CD pain imposes a heavy burden on patients and the healthcare system, and the current treatment strategies may be insufficient to address this unmet need. Therefore, there is a need to explore new and innovative solutions for CD pain management.

CD pain has a complex and multifactorial pathophysiology, involving inflammatory, neuropathic, and psychosocial factors. The pain can be acute or chronic, and it can differ in location, intensity, and frequency [1]. In terms of CD pain, inflammation is a common treatment target, as it triggers the release of mediators that stimulate pain-sensing nerves in the gut. This leads to increased sensitivity of the primary afferent neurons and enhanced pain perception, known as central sensitization [4]. Another factor in CD pain involves the descending pathway from the central nervous system (CNS). This pathway modulates pain transmission by increasing (facilitation) or decreasing (inhibition) input. Consequently, an imbalance in the descending pain modulation towards facilitation can also result in chronic pain. These mechanisms are also involved in central sensitization [4].

In this study, we investigated the efficacy of DRG stimulation as a novel treatment for chronic pain in Crohn's Disease (CD) patients, supported in part by the results of the ACCURATE trial for complex regional pain syndrome and causalgia. This trial revealed that DRG stimulation achieved at least 50 % pain reduction in more than 80 % of the participants after three months of treatment [10]. Additionally, several case reports have investigated the utility of mid-thoracic DRG for persistent post-operative abdominal pain with success in herniorrhaphy and gastric bypass surgeries [11,12].

Fundamentally, the DRG is composed of the somas of sensory neurons that relay sensory information from the periphery to the central nervous system. Afferent peripheral nerves (e.g. A-delta and C fibers) detect pain and send signals through the DRG to the dorsal horn of the spinal cord, where they connect with various neurons before crossing over and ascending via the spinothalamic tracts [13]. Chronic pain disorders are associated with pathophysiologic changes in the DRG, such as alterations in membrane properties, protein expression, and gene expression, that increase neuronal excitability [10]. This increased excitability leads to enhanced pain perception for the individual. DRG stimulation modulates this activity possibly through decreased action potential propagation at the T-junction, which reduces pain [14].

This procedure involved us percutaneously placing stimulator leads near the target DRGs, at the spinal levels of T10-T12 (Fig. 2). These levels corresponded to the dermatomal distributions for the patient's primary regions of pain and the suspected areas of disease. The patient had right-sided abdominal pain, corresponding to the T10-T12 nerve distribution of the midgut, which includes the distal duodenum, jejunum, ileum, cecum, ascending colon and proximal transverse colon; areas often affected by Crohn's. Most midgut associated gastrointestinal pain is mediated by extrinsic visceral afferent fibers running with their paired sympathetic efferent fibers. These afferent fibers retrogradely travel from the intestines through the superior mesenteric plexus, along a splanchnic nerve into the sympathetic trunk and a ventral ramus, before finally separating from the efferent pathway by following the dorsal root into the dorsal root ganglion, where its nerve cell body is located [15]. They then synapse in the dorsal horn of the spinal column at the T10-T12 levels. Our patient had good relief from the T12 DRG lead, but did not experience any relief from the T10 lead. Therefore, for the permanent implant, the team proceeded with T11/T12 lead placement. The patient had sustained pain relief for at least 3 months without any complications related to the device (Fig. 3).

Traditional spinal cord stimulation (SCS) has been the mainstay of neuromodulation technology for decades and has demonstrated moderate efficacy against chronic pain. However, due to the proximity of sensory fibers within the dorsal column to the electrode's area of influence, pain relief is often accompanied/replaced by paresthesia [10]. The advantage of DRG stimulation is that it can more accurately and effectively target therapy to specific spinal levels, by utilizing basic anatomy and stimulating the DRG directly at the relevant spinal segments [16].

## 4 Conclusion

Pain management for acute CD flares is a challenging and incompletely resolved issue. Crohn's Disease causes significant physical, psychological, and financial distress for the patients, as well as a high cost for the healthcare system [[1], [2], [3]]. Therefore, maximizing effective treatment options is essential. DRG stimulation is a novel technique that was approved in 2016 for chronic pain conditions. However, this is the first reported case in literature of using DRG stimulation to treat a patient with severe CD related pain. Although the patient did not achieve complete pain relief, she experienced remarkable functional improvement, including ability to eat, sleep, and get out of bed. She also reported improved mood and overall quality of life.

We aimed to provide a cost-effective treatment that would reduce the patient's pain, enhance her oral food intake and improve her overall quality of life, compared to other long-term treatment options. Chronic pain is a complex and subjective phenomenon that cannot be fully captured by pain scores alone, though they have been traditionally used as a main outcome measure. Therefore, it is important to use a holistic approach to evaluate the effectiveness of the treatment [17]. Moreover, cost-effectiveness studies such as the ACCURATE trial suggest that DRG stimulation is superior to other traditional treatment modalities such as SCS in the long-term, despite the higher initial costs, the quality-of-life benefits may outweigh the cost difference. Additionally, the original battery used in the DRG systems has been replaced with more efficient and longer-lasting technology [16,18].

We hope that this case study will contribute to the advancement of research and treatment options for chronic pain using DRG stimulation, in the context of CD or other chronic abdominal pain conditions.

## Declaration of competing interest

The authors declare that they have no known competing financial interests or personal relationships that could have appeared to influence the work reported in this paper.
